# Internet-Based Cognitive Behavioral Therapy for Informal Caregivers: Randomized Controlled Pilot Trial

**DOI:** 10.2196/21466

**Published:** 2021-04-07

**Authors:** Ieva Biliunaite, Evaldas Kazlauskas, Robbert Sanderman, Inga Truskauskaite-Kuneviciene, Austeja Dumarkaite, Gerhard Andersson

**Affiliations:** 1 Department of Behavioural Sciences and Learning Linköping University Linköping Sweden; 2 Center for Psychotraumatology Institute of Psychology Vilnius University Vilnius Lithuania; 3 Department of Health Psychology University Medical Center Groningen University of Groningen Groningen Netherlands; 4 Department of Psychology, Health & Technology University of Twente Enschede Netherlands; 5 Department of Biomedical and Clinical Sciences Linköping University Linköping Sweden; 6 Department of Clinical Neuroscience Karolinska Institutet Stockholm Sweden

**Keywords:** caregiver burden, informal caregivers, internet intervention, cognitive behavioral therapy, eHealth, mHealth

## Abstract

**Background:**

Caregiving for a family member can result in reduced well-being for the caregiver. Internet-delivered cognitive behavioral therapy (ICBT) may be one way to support this population. This is especially the case for caregivers in countries with limited resources, but high demand for psychological services.

**Objective:**

In this study we evaluated the effects of a therapist-guided 8-week-long ICBT intervention for informal caregivers.

**Methods:**

In total, 63 participants were recruited online and randomized either to the intervention or to the wait-list control group. The main study outcome was the Caregiver Burden Inventory (CBI). Secondary outcomes included measures of caregiver depression, anxiety, stress, and quality of life.

**Results:**

Moderate between-group effect sizes were observed for the CBI measure, in favor of the intervention group, with a Cohen *d*=–0.70 for the intention-to-treat analysis. Analyses of the subscales of the CBI showed significant reductions on the subscales of Development and Physical Health. Moderate reductions were found for depression and anxiety scores as indicated by the Patient Health Questionnaire-9 (PHQ-9) and Generalized Anxiety Disorder-7 (GAD-7) scores. Large between-group effects were observed for reduction in stress and increase in quality of life as indicated by the Perceived Stress Scale-14 (PSS-14), The Brunnsviken Brief Quality of Life Scale (BBQ), and The World Health Organization-Five Well-Being Index (WHO-5). In addition, participants experienced little to no difficulty in using the program and were mostly satisfied with the intervention’s platform and the choice of content.

**Conclusions:**

This is the first internet intervention study for informal caregivers in Lithuania. The results suggest that therapist-guided ICBT can be effective in reducing caregiver burden, anxiety, depression, stress, and improving quality of life.

**Trial Registration:**

ClinicalTrials.gov NCT04052724; https://clinicaltrials.gov/ct2/show/NCT04052724

## Introduction

Because of an increase in longevity and a decrease in fertility, it has been suggested that in the future more individuals will be involved in taking care of the elderly than raising children [1]. Approximately one-third of the population could be described as informal caregivers as they look after someone in their close environment: individual(s) who are either physically or mentally ill, or experience difficulties due to old age [2]. Over the course of the history, women were more often expected to take up caregiving tasks within the family context [3]. Today, caregiving is still more prevalent among women [2]. Across Europe, informal caregiving is also more common among those aged between 50 and 59 as well as among non-employed or religious individuals [2]. Informal caregivers are becoming increasingly relied on for managing health care and societal costs for the elderly and chronically ill [4]. Yet, caregivers themselves are often faced with many challenges which puts them at risk for their own well-being.

Caregiving tasks and intensity vary greatly based on the condition of care recipients, available resources, and a combination of many other factors. Accordingly, caregiving might affect individuals differently too. For example, it has been shown that informal caregiving can bring satisfaction and fulfilment [5], especially if appraised positively [6]. However, this is only one side of the coin. It has been previously found that caregivers, in comparison to noncarers, experience reduced mental well-being [7], chronic stress, and increase in depressive symptoms [8]. Such a combination of physical, psychological, financial, and social demands of caregiving can be overall referred to as caregiver burden [3]. According to this definition, caregiver burden is viewed as a multidimensional experience that affects several aspects of caregiver’s life. Hence, despite the positive consequences, informal caregiving can also have a negative effect on the informal caregiver’s physical and emotional well-being.

Intercultural differences exist in the type of support that informal caregivers receive across Europe [9]. For example, caregivers in the Northern countries, such as Sweden or Denmark, tend to receive tax-funded professional help, and therefore spend on average less time for caregiving duties (approximately 2-3 hours per week) [10]. Contrary to this, caregivers in Southern European countries might be required to provide 24-hour support [10]. These are rather drastic cross-country differences that might in turn have a different effect on caregiver’s overall well-being and experience of burden. Apart from availability of formal support, countries differ in other factors such as traditions and societal expectations toward care provision [3]. It is therefore important to consider specific cultural context when developing support services, and in the present context psychological services delivered via the internet.

Because of the increasing numbers of informal caregivers and evidence of their experienced burden, recent years have seen a rise in research studies investigating ways to improve their well-being. Different types of support interventions could be distinguished: interventions providing education and information, interventions providing practical support via respite services for the caregiver, and interventions providing psychological support [3]. Interventions in the latter group are of particular interest because they target informal caregiver well-being. Specifically, these interventions aim at supporting caregivers in their emotion management as well as problem-solving skills, and hence improving their overall quality of life.

Apart from the traditional, face-to-face format, psychological support interventions are being increasingly offered online. One of the benefits of internet interventions is that it can reach a wide range of individuals, even in remote places [11]. This is a very important benefit as opposed to the face-to-face format, especially in the context of the current COVID-19 pandemic, which is having an impact not only on individual mobility, but also on accessibility of various support services. Regarding internet intervention programs for informal caregivers, differences can be observed in both the mode of delivery and the content of programs [12]. To give an example, some programs provide video materials, whereas others mainly rely on text. In addition, some programs offer human support via professional feedback, whereas others offer opportunities for peer support. Despite differing formats of such interventions, based on the current findings there is some evidence that internet interventions for informal caregivers can be effective in improving their well-being [13]. At the same time, despite the emergence of internet intervention studies for informal caregivers, further high-quality research is encouraged [14].

Internet-delivered cognitive behavioral therapy (ICBT) is one of the treatment formats that now has been tested in various populations with success rate similar to that of face-to-face therapy [15]. Few attempts have been previously made to implement ICBT for informal caregivers (eg, [16]). However, in most cases treatments were targeted to a specific group of caregivers. For example, caregivers of individuals with dementia, cancer, or other disorders. Hence, there is little existing knowledge about the effectiveness of transdiagnostic ICBT for informal caregivers. Transdiagnostic treatments target common mechanisms observed in various psychiatric disorders, instead of focusing on one specific disorder. Such treatments are especially useful in addressing comorbidity. To give an example, the same intervention program could be used by individuals with stress and anxiety as well as depression symptoms. Therefore, if effective, transdiagnostic ICBT could be applied for a wide range of informal caregivers.

Considering the need for further research studies, the growing number of caregivers, and the need for accessible interventions, the aim of this study was to develop and evaluate the effectiveness of an internet-based, therapist-guided ICBT intervention in reducing caregiver burden. Caregiver burden was chosen as a focus for the intervention because current research findings regarding the effectiveness of internet intervention in reducing caregiver burden are inconclusive [6]. The intervention was targeted at informal caregivers in Lithuania and was a set up as a pilot randomized controlled trial. A decrease in the caregiver burden was defined as the primary goal with reduction in depression, anxiety, and stress as well as increase in quality of life as secondary goals. Results of this study will provide information about the acceptability and effectiveness of such interventions in a unique population where the demand for such services is high [17].

## Methods

### Design

**Figure 1:**
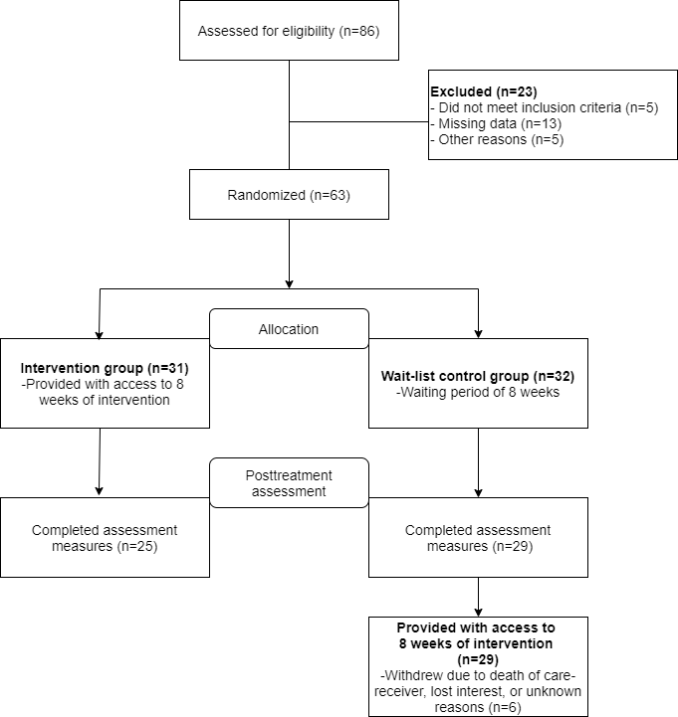
Flowchart of the participant recruitment to the randomized controlled pilot trial.

A 2-armed randomized controlled trial was conducted online in Lithuania with participants recruited from the general population. Participants were randomly allocated to either an 8-week internet treatment or a wait-list control condition. Measures were collected online and administered at 2 points in time: at the start of the treatment (pretreatment) and at the end of the treatment (posttreatment). In addition, 4 weeks after the start of the intervention, participants were contacted for a short telephone interview. The main purpose of this conversation was to provide participants with an opportunity to ask questions about the use of the treatment as well as to raise any related concerns. Control group participants received the same treatment once the initial treatment phase had finished (posttreatment). A flowchart representation of the study is presented in Figure 1. Ethical approval for this study was received from the Vilnius University Psychology Research Ethics Committee (08-07-2019 No. 26). The trial was registered in ClinicalTrials.gov (registration number NCT04052724) and reported in accordance with the CONSORT-EHEALTH checklist (Multimedia Appendix 1).

### Participants

Eligible participants were able to register from September 2019 to October 2019. Recruitment was conducted in various forms. The study was announced by the Vilnius University press release and posted on the Vilnius University website, Facebook, and other social media. Additionally, invitation to participate in the study was disseminated among the health care institutions, social services, and patient organizations targeted to reach informal caregivers. The study publicity campaign reached national media, and the study was covered by national TV and radio programs. In addition, information about the study was posted in national and regional press. After registration, individuals who filled in all the required measures online were contacted for a structured interview over telephone, after which eligibility to participate in the study was finalized.

To participate in the study, individuals had to have access to the internet via a computer or any other compatible device (eg, smartphone or tablet) as well as the ability to use it throughout the duration of the intervention. Eligible participants had to have a score of 24 or more on the Caregiver Burden Inventory (CBI) [18], be 18 years old or over, and be fluent in comprehending, writing, and reading Lithuanian language. The intervention was not tailored to any specific group of caregivers (eg, those caring for patients with dementia). Individuals were not eligible to participate in the study if they were experiencing severe physical or mental health problems, alcohol addiction, other severe traumatic events, suicide risk, severe interpersonal violence, or their care receiver had a life expectancy below or approximately around 6 months.

Overall, 86 individuals expressed interest to participate and provided informed written consent. Of these, 13 did not fill in all the required questionnaires. One participant was excluded as we were not able to reach the person for screening. Eligibility of the remaining 72 participants was assessed via phone interviews. Mini International Neuropsychiatric Interview Version 5 [19] was translated into Lithuanian language by the research group and was used in phone interviews for additional assessment of inclusion criteria. Following interviews, 9 participants were excluded due to either not meeting inclusion criteria (n=5) or losing interest in participation (n=3). One remaining application was removed, because the participant had registered twice. We have made an exception regarding cut-off scores for CBI: 1 participant with a score of 21 was included in the study because the individual expressed feeling burdened as well as wished for support during the phone interview. Hence, 63 participants were randomized to either the intervention (n=31) or the wait-list control group (n=32). Randomization was conducted by an independent researcher not involved in the trial and performed according to a 1:1 ratio. The website for generation of random numbers [20] was used. Randomization procedure was also used for randomly allocating therapists to participants.

All the included participants were requested about the condition of their care receiver. As there would be several small groups among our sample regarding different medical conditions of the care receivers, we chose not to further categorize these data. Moreover, many caregivers indicated that their care receiver experiences several comorbidities, which would make it difficult to categorize the data. Nevertheless, some of the more common health conditions of the care receivers were dementia and frailty due to old age.

### Measures

#### Primary Outcome Measure

##### Caregiver Burden Inventory

The CBI [18] was chosen as it views caregiver burden as a multidimensional experience. The CBI comprises 24 items that are distributed within 5 areas: development, physical health, time dependency, emotional health, and social relationships. Response options are presented on a 5-item Likert scale ranging from 0 (Never) to 4 (Nearly always). All areas have 5 questions dedicated to them with the exception of physical health, which has 4. The total score of the CBI is summed up and ranges from 0 to 96, with higher scores indicating higher levels of burden. Reliability coefficients for each of the subscales of the CBI were previously shown to be high: development (α=.87), physical health (α=.86), time dependency (α=.85), emotional health (α=.81), and social relationships (α=.69) [21]. In this sample, Cronbach α for each of the subscales was high: development (α=.85), physical health (α=.80), time dependency (α=.89), emotional health (α=.83), and social relationships (α=.81). Cronbach α for CBI altogether in this sample was also high (α=.87). The CBI has been translated and previously applied in research studies in several different countries (eg, [22,23]) as well as for specific caregiver groups. In our study, the CBI was translated into Lithuanian language by the research group.

#### Secondary Outcome Measures

For these measures existing official Lithuanian language translations were used.

##### Patient Health Questionnaire-9

The Patient Health Questionnaire-9 (PHQ-9) [24] comprises 9 questions aimed at evaluating depressive symptoms. Response options are presented on a 4-item Likert scale ranging from 0 (Not at all) to 3 (Nearly every day). Higher scores indicate higher symptom severity. This questionnaire is widely used in research studies, due to easy administration and very good psychometric properties (α=.89) [24]. In this sample, Cronbach α for PHQ-9 was high (α=.82).

##### Generalized Anxiety Disorder-7

The Generalized Anxiety Disorder-7 (GAD-7) questionnaire [25] is used for evaluating symptoms of generalized anxiety disorder. This questionnaire consists of 7 questions. As with PHQ-9, answers for this questionnaire are distributed on a 4-item Likert scale ranging from 0 (Not at all) to 3 (Nearly every day). Higher score indicates higher levels of anxiety. This measure displays very good psychometric properties (α=.92) [25]. In this sample, Cronbach α for GAD-7 was high (α=.87).

##### Perceived Stress Scale-14

The Perceived Stress Scale-14 (PSS-14) [26] is a 14-item questionnaire that contains questions measuring stress on a 5-point Likert scale ranging from 0 (‘Never’) to 4 (‘Very Often’) [26]. Items on the PSS-14 touch upon aspects such as feeling of nervousness and ability to cope as experienced over the last 4 weeks. Higher scores on PSS-14 indicate higher levels of stress. This measure has previously shown good psychometric properties with Cronbach α ranging from .75 to .89 [27]. In this sample, Cronbach α was found to be high (α=.85).

##### Brunnsviken Brief Quality of Life Scale (BBQ)

The Brunnsviken Brief Quality of Life Scale (BBQ) [28] is a questionnaire developed for evaluating quality of life for both clinical and nonclinical samples. It contains 12 statements that cover topics such as creativity, leisure time, and view on oneself. Responses are distributed on a 5-point Likert scale ranging from 0 (Strongly disagree) to 4 (Strongly agree). Higher scores on this measure indicate higher quality of life. Psychometric properties of BBQ are good (α=.76) [28]. The BBQ in our sample showed good internal consistency (α=.87).

##### The World Health Organization-Five Well-Being Index

The World Health Organization-Five Well-Being Index (WHO-5) [29] is a widely used short questionnaire that was developed by the World Health Organization [30] and has shown to be a valid and reliable measure of overall well-being (α=.88) [29]. This questionnaire contains 5 statements regarding an individual’s well-being over the last 2 weeks. Each of the statements is evaluated using a 6-item Likert scale ranging from 0 (At no time) to 5 (All the time). Higher scores indicate higher well-being. The internal consistency for WHO-5 in our sample was acceptable (α=.76).

#### Baseline Measures

##### Life Events Checklist

The Life Events Checklist (LEC) [31] was developed at the National Center for Post-Traumatic Stress Disorder to screen for potentially traumatic events in a respondent’s lifetime. In this study, we used 19-item version that has been adapted to the Lithuanian cultural context [32]. This questionnaire has acceptable psychometric properties (κ=0.61) [33]. In our sample, the internal consistency for LEC was acceptable (α=.78).

##### The Alcohol Use Disorders Identification Test (AUDIT)

The Alcohol Use Disorders Identification Test (AUDIT) [34] is a 10-item instrument for assessing alcohol consumption and indicating its consequences on the well-being of an individual. Each statement is evaluated by choosing an answer from either 5 or 3 different response options. This instrument has been a subject of extensive evaluation and has consistently displayed good psychometric properties with a median reliability value of 0.82 for non-English versions [35]. The AUDIT in our sample displayed acceptable internal consistency (α=.73).

### Intervention

**Figure 2:**
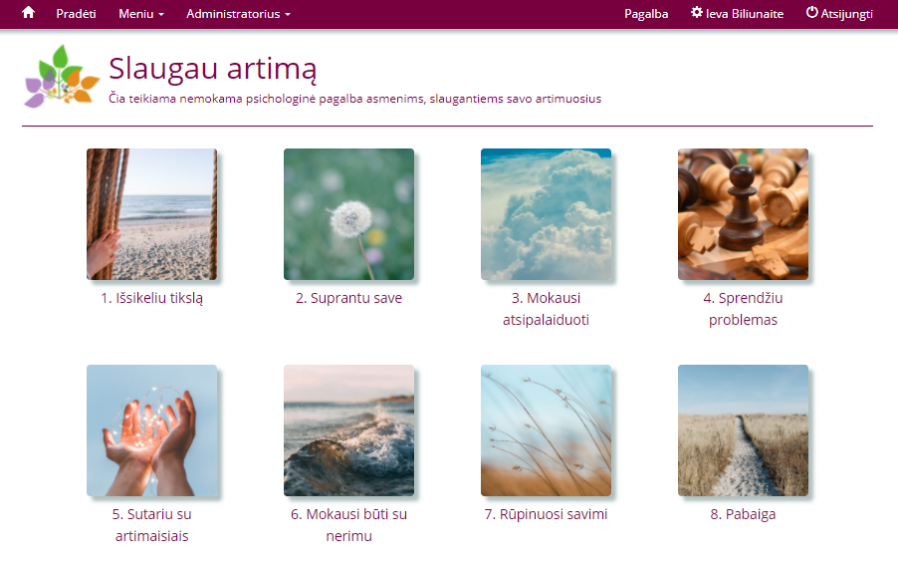
Screenshot of the eight intervention modules as presented to participants after logging in.

The intervention was an ICBT program consisting of 8 modules, each dedicated to 1 theme. The themes were (chronologically): introduction, thoughts, stress, and relaxation, problem solving, communication, anxiety, behavioral activation, and maintenance. The content was mainly presented in the form of written text with the exception of relaxation instructions, which participants could choose to either read or listen to. See Figure 2 for a screenshot of the 8 modules in the program provided to participants after logging in. In Lithuanian language, the name of the program was *Slaugau artimą*, which if translated literally means, “I take care of my close one.” The intervention content was partly adapted from materials previously used in other ICBT studies (eg, [36-38]). The choice of content, examples, and exercises of the intervention were carefully selected and adopted for the target population grounded on the practices and guidelines in the field of informal caregiving (eg, [3]). A short description of the 8 modules as well as a list of exercises for each module is presented in Table 1.

Participants were provided access to 1 new module every week over the 8 weeks of intervention, starting from module 1. Once accessible, modules remained available throughout the duration of the treatment. The basic structure of the modules is very similar throughout the intervention: each module starts with psychoeducation, followed by one or few case examples which are then followed by several exercises. At the end of each module, participants were provided with suggestions about which exercises they should practice further that week. In this way participants were encouraged to learn and apply gained knowledge in managing maladaptive thoughts or behaviors in their own situation. In cases of lack of clarity, participants were able to contact their assigned therapist via a secure internal messaging function on the platform. Therapists monitored participants’ questions and provided feedback on completed exercises daily via the intervention’s platform.

**Table 1:** Description of intervention content.

Module (length)	Goals of the module	Exercises
Introduction (1089 words)	Instruct about how to use the intervention Introduce main principles of cognitive behavioral therapy Inform about what participants can expect throughout the treatment Encourage to formulate individual goals for the duration of the program	Writing exercise I—Describing emotionally difficult caregiving-related situation and further reflecting on thoughts, behavior, and short- and long-term consequences. Writing exercise II—Setting goals for the treatment
Thoughts (1682 words)	Discuss how thoughts impact on one’s well-being Introduce the concept of automatic thoughts Instruct about how to practice recognizing automatic thoughts Introduce the most common cognitive distortions	Thinking of own thought patterns and cognitive distortions Thought Change Record—participants use a predefined structure to evaluate their situation, automatic thoughts, emotions, rational response, and outcome
Stress and relaxation (2426 words)	Define stress Discuss what effect stress can have on well-being Discuss how one can reduce stress in daily life Familiarize with relaxation methods	Writing exercise—Thinking and describing which caregiving-related situation(s) causes most stress Thinking of ways to increase inner resources and lower mental or physical load Planning when to engage in pleasurable activities Listening and practicing short meditations
Problem solving (1524 words)	Encourage to reflect on own problem-solving strategies Familiarize with different coping strategies Emphasize helpful and harmful coping strategies Learn about factors that interfere with problem solving	Writing exercise—Thinking and describing strategies that an individual uses to deal with problems Based on provided theory and examples, reflecting once more on problem-solving strategies Solving own problem(s) a using step-by-step approach
Communication (2635 words)	Discuss possible changes in communication after one becomes a caregiver Discuss possible ways of maintaining or improving communication with people in a close environment	Reflecting on changes in communication after becoming a caregiver Step-by-step structure from previous module for solving communication problems Perspective taking exercise—Evaluating difficulties in communication from a different point of view Writing exercise—Using writing to express own emotions
Anxiety (2090 words)	Discuss what anxiety is and what effect it can have on well-being Discuss ways in which anxiety could be reduced	Reflecting on sources of worry in own situation Exercise “Balcony”—Evaluating a worrisome situation by observing it from the distance Listening and practicing short meditations
Behavioral activation (1338 words)	Emphasize the importance of personal time and small rewards Encourage to spend time for own needs	Reflecting on behavior and activities that are not useful Thinking of how one can find more time and opportunities for pleasant activities
Maintenance (906 words)	Summarize most important points Provide with tips for maintaining gained knowledge Encourage to reflect on previously formed goals and goals for the future Farewell	Reflecting on what was the most useful throughout the program and which knowledge or exercises one can also apply in the future Reflecting on initial goal and progress throughout the program

### Therapists

Three Master’s in Clinical Psychology program students trained to deliver this intervention under supervision and 3 clinical psychologists were involved as therapists in the study. The main role of the therapists was to answer participant questions as well as to provide feedback on completed modules via a secure messaging system in the intervention’s platform [39]. Weekly scheduled and on-demand supervision meetings for therapists were conducted and lead by one of the experienced clinical psychologists (AD) in the study. These supervision meetings varied in length between 1 and 2 hours.

### Procedure

Upon opening the study website, participants were provided with information regarding inclusion and exclusion criteria as well as the registration process, management, and research team. After participants provided informed consent on a secure study website, they were asked to fill in the screening questionnaires. Participants were then invited for a phone interview. Following phone interviews, the final decision about participation in the study was jointly discussed and agreed upon by the 3 coauthors (IB, IT-K, and AD). Decision about exclusion or inclusion in the study was communicated to the participants in a span of a few days. After randomization, participants in the intervention and wait-list control group were provided with information about the start of the intervention. Participants in the waiting-list control group were also explained that they will be able to receive access to the same treatment after the intervention group is finished.

A secure online platform (*Iterapi* [40]) was used for communication between therapists and participants, distribution of program materials, and collection of assessments. Participants’ personal information was anonymized by assigning each participant a code, which participants then used for logging into the program. Included participants were also able to extend their data security by receiving a code (one-time password) to their phone number, which had to be entered upon every logging in, after entering their self-generated password.

At the start of the intervention, all participants received an email containing their username as well as personalized link for creating their own password. Throughout the duration of the program, every Thursday, participants received an email stating availability of the new material. Participants who did not view a particular week’s materials or have not conducted exercises were sent 1 weekly reminder. Reminder contained a predefined short encouraging message and was sent on Mondays, by therapists to participants in own groups.

### Statistical Analysis

Analysis was performed using IBM SPSS Statistics (version 25). The significance level was set at .05. Independent samples *t* test and Fisher exact test were used for investigating possible differences among participants in the 2 groups at baseline. Data collected at posttreatment were treated according to the intention-to-treat (ITT) principle. Following ITT all participants were analyzed according to their treatment assignment included in the analysis [41]. This method was chosen as it preserves the integrity of the randomization and minimizes the risks of bias that could occur due to the differences in groups following attrition or nonadherence [42]. For this purpose, a multiple imputation procedure was chosen considering that data were missing at random (MAR) [43]. According to MAR, missingness depends on the observed data, where incomplete values are replaced by values that are based on the complete data [44]. In our study MAR was considered, as no clear patterns in the missing data regarding pretreatment scores or demographic variables were identified. During the multiple imputation procedure, 20 simulations were imputed in a sequence. Because it is practically difficult to be fully confident that data are missing at random, complete case analyses were also performed.

Descriptive statistics were used for evaluating attrition and overall satisfaction with the program. Regarding main outcomes, effect sizes and confidence intervals were calculated for within and between groups. Analysis of covariance (ANCOVA) was conducted to investigate treatment effects at posttreatment for primary and all secondary measures with baseline scores entered as covariates [45].

The Jacobson and Truax [46] method was used for calculating Reliable Change Index (RCI) and investigating clinical significance of change in primary outcome at posttreatment. Because population norms were not available, criteria *a* was calculated, resulting in a cut-off point of 28.8. According to this, individuals who scored below 28.8 points at posttreatment should fall outside of the dysfunctional population and be considered recovered [46]. As for reliability of the scores, RCI values were calculated. In cases where a positive reliable change was achieved, participants were deemed improved. In turn, negative reliable change scores were used to determine deterioration. In the current sample, participants had to obtain a reduction of 8.8 scores on CBI at postassessment for a positive reliable change to be achieved.

## Results

### Participants

Most of the recruited participants were female (57/63, 90%) caring for either their elderly mother or father (44/63, 70%). Most participants had provided care for either 1-4 years (28/63, 44%) or more than 4 years (25/63, 40%). Most caregivers were spending 5-7 days per week (54/63, 86%), and either 3-7 (24/63, 38%) or 12 or more hours (23/63, 37%) per day providing care. In this respect, the sample could be described as consisting of high-intensity, long-term carers. Demographic characteristics of the sample are presented in Table 2. No pretreatment differences regarding demographic or outcome measures between participants randomized to treatment and wait-list control group were detected.

**Table 2:** Sociodemographic characteristics of the sample at baseline.

Participant characteristics			Overall (n=63)		Intervention group (n=31)	Wait-list control group (n=32)	
Age caregiver (year), mean (SD)			52 (8.4)		54 (7.9)	50 (8.57)	
Age recipient (year), mean (SD)			71 (21.1)		70 (23.13)	72 (19.28)	
Gender (female) caregiver, n (%)			57 (90)		28 (90)	29 (91)	
Gender (female) recipient, n (%)			44 (70)		20 (65)	24 (75)	
**Relation receiver, n (%)**							
	Husband/wife/partner	8 (13)		5 (16)			3 (9)
	Father/mother	44 (70)		20 (65)			24 (75)
	Other	11 (17)		6 (19)			5 (16)
**Time caring (months), n (%)**							
	<12	10 (16)		6 (19)			4 (13)
	12-48	28 (44)		13 (42)			15 (47)
	>48	25 (40)		12 (39)			13 (41)
**Time week (days), n (%)**							
	1-2	3 (5)		1 (3)			2 (6)
	3-4	6 (10)		3 (10)			3 (9)
	5-7	54 (86)		27 (87)			27 (84)
**Time day (hours), n (%)**							
	3<	9 (14)		5 (16)			4 (13)
	3-7	24 (38)		14 (45)			10 (31)
	8-11	7 (11)		5 (16)			2 (6)
	>12	23 (37)		7 (23)			16 (50)
Residing with care receiver (yes), n (%)			49 (78)		24 (77)	25 (78)	
Individual is the only caregiver (yes), n (%)			31 (49)		18 (58)	13 (41)	
**Highest education level, n (%)**							
	High school or lower	4 (6)		3 (10)			1 (3)
	Professional/vocational training	17 (27)		9 (29)			8 (25)
	College or applied science education	6 (10)		2 (6)			4 (13)
	University degree	36 (57)		17 (55)			19 (59)
**Marital status, n (%)**							
	Single	12 (19)		7 (23)			5 (16)
	Married/partner	39 (62)		18 (58)			21 (66)
	Divorced/widowed or other	12 (19)		6 (19)			6 (19)

### Attrition

Participants were regarded as dropouts if the posttreatment measures were missing. Independent samples *t* test and Fisher exact test were performed for investigating differences between dropouts and completers. No differences were detected between the groups regrading demographic characteristics or scores on outcome measures.

At posttreatment, out of 31 participants in the intervention group, 25 have filled in the measures (81%), yielding a dropout rate of approximately 19%. At the same time, 29 out of 32 participants have filled in the measures in the wait-list control group (91%). Overall, posttreatment measures were collected from 54 participants (86%), which indicated a dropout rate of 14%. Following the intervention, out of 32 participants in the wait-list control group, 23 had filled in the measures (72%), showing a dropout rate of approximately 28%. In total, the overall dropout rate (ie, both groups combined) after the intervention period ended was 24% (15/63).

We were not able to reach all the participants who did not fill in the posttreatment measures. From participants that we did manage to reach, there were 2 main reasons for ceased participation: losing interest in participation (n=3) or death of care receiver (n=3).

### Main Outcomes

Within- and between-group effect sizes were calculated for both completers and ITT sample. We found moderate within- and between-group effect sizes for the CBI measure (Table 3). Besides, moderate to large between- and small to large within-group effect sizes for secondary outcome measures were observed. Effect sizes for the completers were higher than those for ITT, indicating that the ITT result should be the one to consider. Further, only the ITT results will be reported. Completer results are provided in Multimedia Appendix 2.

The ANCOVAs were performed to determine differences between the control and intervention group on the posttreatment scores when controlling for the pretreatment scores. For primary and all secondary measures, significant effects of group on posttreatment scores were found in the ITT sample—CBI: *F*_1,60_=5.39, *P*=.02; PHQ-9: *F*_1,60_=6.12, *P*=.01; GAD-7: *F*_1,60_=8.24, *P*=.004; PSS-14: *F*_1,60_=13.56, *P*<.001; BBQ: *F*_1,60_=10.88, *P*=.001; and WHO-5: *F*_1,60_=10.7, *P*=.001. The ANCOVAs were also conducted to investigate changes over 5 subscales of the CBI separately at posttreatment. In the ITT sample significant changes have been detected for the reduction in postassessment scores in the subscales of Development and Physical Health, *F*_1,60_=6.99, *P*=.008 and *F*_1,60_=5.5, *P*=.02, respectively. No significant changes were observed for the remaining 3 subscales—Time Dependency: *F*_1,60_=0.25, *P*=.62; Emotional Health: *F*_1,60_=2.05, *P*=.15; and Social Relationships: *F*_1,60_=2.67, *P*=.10.

**Table 3:** Means, standard deviations, and effect sizes (Cohen *d*) with confidence intervals.

Measures and condition			Intention to treat, mean (SD)				Completers, mean (SD)			Effect size (95% CI)				
			Pretreatment (n=63)		Posttreatment (n=63)		Pretreatment (n=63)		Posttreatment (n=54)			Within-group prepost		Between-group posttreatment
**CBI^a^**														
	Intervention	51.94 (12.79)		46.39 (13.78)		51.94 (12.79)		46.28 (13.92)			0.41 (–0.09 to 0.92)		–0.7 (–1.2 to –0.19)	
	Wait-list	55.84 (12.44)		56.99 (16.22)		55.84 (12.44)		57.34 (16.1)			0.13 (–0.36 to 0.62)			
**PHQ-9^b^**														
	Intervention	9.32 (4.42)		8.23 (4.79)		9.32 (4.42)		7.84 (4.78)			0.24 (–0.27 to 0.73)		–0.69 (–1.19 to –0.17)	
	Wait-list	10.41 (5.36)		12.05 (6.2)		10.41 (5,36)		12.14 (6.28)			–0.28 (–0.77 to 0.21)			
**GAD-7^c^**														
	Intervention	8.19 (4.34)		6.83 (4.28)		8.19 (4.34)		6.48 (4.12)			0.32 (–0.19 to 0.81)		–0.74 (–1.24 to –0.22)	
	Wait-list	9 (4.95)		10.77 (6.14)		9 (4.95)		10.9 (6.25)			–0.32 (–0.81 to 0.18)			
**PSS-14^d^**														
	Intervention	27.03 (5.97)		21.83 (6.84)		27.03 (5.97)		21.08 (6.21)			0.81 (0.28 to 1.32)		–1.06 (–1.57 to –0.52)	
	Wait-list	29.28 (7.89)		30.07 (8.58)		29.28 (7.89)		30.69 (8.3)			–0.10 (–0.58 to 0.40)			
**BBQ^e^**														
	Intervention	45.26 (22.32)		56.69 (25.04)		45.26 (22.32)		58.44 (25.18)			–0.48 (–0.98 to 0.03)		0.8 (0.28 to 1.30)	
	Wait-list	41.75 (23.19)		37.77 (22.18)		41.75 (23.19)		37.14 (21.78)			0.18 (–0.32 to 0.66)			
**WHO-5^f^**														
	Intervention	34.97 (12.04)		48.66 (19.72)		34.97 (12.04)		49.92 (19.8)			–0.8 (–1.3 to –0.3)		0.85 (0.32 to 1.35)	
	Wait-list	32.75 (16.52)		32.72 (17.84)		32.75 (16.52)		32.55 (17.75)			0 (–0.50 to 0.49)			

^a^CBI: Caregiver Burden Inventory.

^b^PHQ-9: Patient Health Questionnaire-9.

^c^GAD-7: Generalized Anxiety Disorder-7.

^d^PSS-14: Perceived Stress Scale-14.

^e^BBQ: The Brunnsviken Brief Quality of Life Scale.

^f^WHO-5: The World Health Organization-Five Well-Being Index.

### Clinically Significant Change and RCI of Caregiver Burden

Results regarding RCI for the ITT sample are presented in Table 4. McNemar test was performed to test whether the difference between participants in the 2 groups regarding positive reliable change is significant. An exact McNemar test result indicated that significantly more participants in the intervention group as opposed to the wait-list control group have achieved a positive reliable change for CBI scores at postassessment (*P*<.001). Regarding clinical significance, 2 (6%) participants in the intervention group scored below the cut-off score of 28.8. These participants were deemed as recovered because they achieved a clinically significant change. In the control group, 1 (3%) participant was found to achieve a clinically significant change.

**Table 4:** RCI of participants in the ITT sample.

Group	RCI^a^ for CBI^b^ scores		
	Positive RCI	No change	Negative RCI
Intervention (n=31), n (%)	14 (45)	13 (42)	4 (13)
Wait-list (control; n=32), n (%)	3 (9)	23 (72)	6 (19)

^a^RCI: Reliable Change Index.

^b^CBI: Caregiver Burden Inventory.

### Participant Evaluation of the Intervention

Overall, 56% (14/25) of participants who filled in posttreatment measures in the intervention group indicated that throughout the program their well-being had improved. Most participants also indicated that using the program was either easy (13/25, 52%) or very easy (6/25, 24%) and was either useful (12/25, 48%) or very useful (7/25, 28%). Participants were also positive about the opportunity to contact and receive feedback from a therapist, with 44% (11/25) indicating that this opportunity was useful, while 28% (7/25) of participants rated it as very useful. Lastly, information and tasks in the program were mostly rated as useful (10/25, 40%) or very useful (9/25, 36%).

Almost half of the participants (12/25, 48%) indicated that all the modules in the program were useful and none (0/25, 0%) noted that modules were not useful. The highest ranking in terms of usefulness was module 7 (“behavioral activation”; 16/25, 64%). The second most useful was module 6 (“anxiety”; 14/25, 56%). The modules “stress and relaxation” and “problem solving” were also rated as very useful by more than half of the participants (13/25, 52%). Module 1 ("introduction") was rated as the least useful (5/25, 20%).

## Discussion

### Principal Findings

The aim of this study was to evaluate the effectiveness of an internet-based therapist-guided program for reducing caregiver burden. Moderate between- and within-group effect sizes were found in reducing caregiver burden in the intervention group. This is a promising finding keeping in mind that previous face-to-face studies have achieved small to moderate between-group effect sizes (*d*=0.09-0.23) [47]. Our results also revealed that out of 5 caregiver-burden components, significant posttreatment reductions were observed for the subscales of Physical Health and Development. The latter subscale consists of items regarding missing out on life, emotional tiredness, and limited social life. In turn, the Physical Health subscale questions sleep disturbances, worsened health, and tiredness. It could be argued that behavioral and cognitive components of the intervention, such as time scheduling, thought diary, stress management exercises, and relaxation methods, helped participants to address these areas the most. To our knowledge, there is only 1 previous study that has reported improvement in these subscales of caregiver burden [48]. This is a study by Spatuzzi and colleagues [48], in which caregivers (of patients with cancer) with low spiritual well-being were compared with those with high spiritual well-being, with the latter found to be experiencing significantly less burden in the Development and Physical Health subscales of CBI. In their study, spiritual well-being was described as one of the core domains in quality of life that also helps in dealing with caregiving tasks. Improvements in quality of life ratings in our sample come in hand with this explanation. However, further investigation is warranted before any conclusions can be drawn.

Moderate to high between-group effect sizes were found on measures of anxiety, depression, and stress as well as increase in quality of life scores with only slight differences between ITT and completer analysis. These findings are in line with existing evidence of internet intervention potential in improving caregiver well-being [14]. We consider this finding of great importance because participants in our sample were found to be highly invested in caregiving: caring on average 5-7 days per week, between 3 and 7 or more than 12 hours per day. The latter could also explain the finding that only 2 participants had clinically recovered and slightly less than half had achieved a positive reliable change in relation to their burden. Caregiver burden, as described by Novak and Guest [18], is a complex experience that compromises emotional, physical, developmental, social, and time-dependency factors. Although our intervention provided participants with tools to deal with negative automatic thoughts, manage stress and anxiety, improve communication, and be attentive to their own needs, it could not change caregiving tasks or the amount of time required for supporting the care receiver. Nevertheless, moderate to high effect sizes found for the primary and secondary measures indicate that internet-delivered CBT (ICBT) can be effective in improving caregiver well-being in Lithuania. This argument is further supported by participant evaluations: more than half of the participants indicated that their well-being improved throughout the duration of the program and almost half noted that all modules were useful and the program was easy to use.

### Limitations and Strengths

One of the main limitations in this study is that we did not control for external help that caregivers were receiving. Although all participants in the study identified themselves as primary caregivers, they could have been receiving various levels of support from other family members or professional workers such as nurses. A second limitation concerns the implementation of the CBI measure and The Mini International Neuropsychiatric Interview (MINI) that were used for the screening purposes. This was the first time both instruments were translated and used in Lithuanian language which could bias the validity and reliability of these measures. Importantly, forward and backward translations were performed independently by the 2 bilingual members (IB and AD) of the research team. Independent versions were then compared and finalized following discussion among the research group members. In addition, translations were presented to a small convenience sample for evaluation of the comprehension of the items. By following such a procedure, we wanted to make sure that translated versions are as accurate as possible while culturally appropriate. We have chosen such an approach due to the pilot nature of the study. We aim to further investigate psychometric properties of these instruments before their implementation in subsequent trials.

Few other limitations must be mentioned. One is that all participants in the study were self-referred which limits the generalizability of our findings and presents the risk of the volunteer bias. Another important limitation is lack of information regarding the long-term effects of the intervention. In addition to the postintervention assessment, follow-up with the participants is highly desirable for investigating if the effects of the intervention were maintained long term. Consequently, it is important to mention that we included 1 participant with a score few points below the initial cut-off point for the CBI. One approach could have been to exclude this individual, but taking into account the pilot nature of the study, we have decided to include the participant and to further reflect on this decision when reviewing inclusion criteria for a prospective larger trial in the future. Lastly, even though positive changes in caregiver well-being were identified, at this point it is not possible to determine which components of the intervention were responsible for this improvement. We aim to further investigate this in the future.

Informal caregivers supporting individuals with a range of support needs were included in this study. For example, there were individuals who were caring for a sibling with a mental disorder as well as their own underage children or elderly parents diagnosed with dementia. For this reason, it could be argued that the intervention did not equally suit everyone’s need. However, our initial idea was to create an intervention that would be suitable to a wide group of caregivers. In that way, independent of the care receiver’s condition, everyone would find something that is applicable to one’s own situation. The fact that we managed to detect improvement in caregiver well-being indicates that such transdiagnostic interventions can be effective. As another strength, we would like to outline the relatively low drop-out rates in our study. We consider the therapist support, weekly reminders, and feedback that participants received from the therapist as possibly the most effective factors in keeping participants engaged with the intervention. Lastly, there were no technical problems with the intervention that could have impacted participant’s overall experience. A full-time IT technician was responsible for managing the technical aspects of the intervention’s platform and thus possible problems would have been handled rapidly.

### Clinical Implications

As briefly outlined previously, informal caregivers represent an increasing part of the society. Their task, even though rewarding, is still demanding. As our results indicate, ICBT can be effective in reducing caregiver burden as well as other negative states such as stress. In turn, it can also be effective in improving the overall quality of life. Importantly, it can reach a wide range of individuals, even the ones in remote locations. Internet interventions as opposed to face-to-face interventions are most likely even of a greater importance currently, considering the COVID-19 pandemic. In addition, for many individuals such a solution could be much more affordable not only time wise, but also financially. As for our target group, informal caregivers in Lithuania, such solutions are of even higher importance considering that psychological support services in general are very limited and not always accessible for all [17]. At this point, we must stress the pilot nature of the study and that our results should be interpreted with caution. Yet, we encourage additional research to further build on this idea and extend our findings in larger randomized trials. Because the numbers of informal caregivers is increasing worldwide and is only considered to further grow in the future, researchers are encouraged to further pursue these ideas in diverse cultural backgrounds, possibly with additional emphasis on the countries in which strong familial norms and little formal support are available to support informal caregivers.

### Conclusion

This study is the first internet intervention study aimed at informal caregivers in Lithuania. The internet-based therapist-guided intervention based on CBT principles was found to be moderately effective in reducing caregiver burden, anxiety, and depressive symptoms. It was also found to be highly effective in reducing stress and improving quality of life for informal caregivers supporting individuals with various care needs. Such an intervention could be of special importance for caregivers who due to time-constraints, geographical, medical, or other reasons are not able to attend face-to-face therapy. Because of close cultural and historical aspects, this experience could further extend to neighboring Baltic countries in the region such as Latvia, where the need for psychological services for the informal caregivers is also high.

## Appendix

Multimedia Appendix 1 CONSORT-EHEALTH checklist.

Multimedia Appendix 2 Results based on completer sample.

## Abbreviations

AUDIT: The Alcohol Use Disorders Identification Test
BBQ: The Brunnsviken Brief Quality of Life Scale
CBI: Caregiver Burden Inventory
CBT: cognitive behavioral therapy
GAD-7: Generalized Anxiety Disorder-7
ICBT: Internet-delivered cognitive behavioral therapy
ITT: Intention-to-treat principle
LEC: Life Events Checklist
MAR: missing at random
PHQ-9: Patient Health Questionnaire-9
PSS-14: Perceived Stress Scale-14
RCI: Reliable Change Index
WHO-5: The World Health Organization-Five Well-Being Index
