# Supplementary material for: Internet-Based Cognitive Behavioral Therapy for Informal Caregivers: Randomized Controlled Pilot Trial
Source: J Med Internet Res. 2021 Apr 7;23(4):e21466. doi: 10.2196/21466 (PMC8060860; doi:10.2196/21466)
Supplement: Multimedia Appendix 2 [file jmir_v23i4e21466_app2.docx]

Multimedia Appendix 1: Results based on completer sample

Main outcomes

Within and between-group effect sizes for completers as well as ITT are presented in the Table 5.

Table 5 *Effect sizes (Cohen’s d) with confidence intervals for completers and ITT*

|  | **Effect size and** CI **based on ITT** | | **Effect size and CI** **based on completer sample** | |
| --- | --- | --- | --- | --- |
| **Measures and condition** | **Within-group pre-post** | **Between group post-treatment** | **Within-group pre-post** | **Between group post-treatment** |
| **CBI** |  |  |  |  |
| Intervention | 0.41 (-0.09 – 0.92) | -0.7 (-1.2 - -0.19) | 0.43 (-0,11-0.95) | -0.73 (-1.27 - -0.17) |
| Wait-list | 0.13 (-0.36 – 0.62) |  | -0.1 (-0.61 – 0.40) |  |
| **PHQ-9** |  |  |  |  |
| Intervention | 0.24 (-0.27 – 0.73) | -0.69 (-1.19 - -0.17) | 0.32 (-0.21-0.85) | -0.76 (-1.31 - -0.2) |
| Wait-list | -0.28 (-0.77 – 0.21) |  | -0.3 (-0.80 – 0.21) |  |
| **GAD-7** |  |  |  |  |
| Intervention | 0.32 (-0.19 – 0.81) | -0.74 (-1.24 - -0.22) | 0.4 (-0.13-0.93) | -0.82 (-1.37 - -0.25) |
| Wait-list | -0.32 (-0.81 – 0.18) |  | -0.34 (-0.84 – 0.17) |  |
| **PSS-14** |  |  |  |  |
| Intervention | 0.81 (0.28 – 1.32) | -1.06 (-1.57 - -0.52) | 0.98 (0.41-1.52) | -1.3 (-1.86 - -0.69) |
| Wait-list | -0.10 (-0.58 – 0.40) |  | -0.17 (-0.68 – 0.33) |  |
| **BBQ** |  |  |  |  |
| Intervention | -0.48 (-0.98 – 0.03) | 0.8 (0.28 – 1.30) | -0.56 (-1.09 – -0.01) | 0.91 (0.34 – 1.46) |
| Wait-list | 0.18 (-0.32 – 0.66) |  | 0.2 (-0.30 – 0.71) |  |
| **WHO-5** |  |  |  |  |
| Intervention | -0.8 (-1.3 - -0.3) | 0.85 (0.32 – 1.35) | -0.94 (-1.48 - -0.37) | 0.93 (0.35 – 1.48) |
| Wait-list | 0 (-0.50 – 0.49) |  | 0.02 (-0.49 – 0.52) |  |
|  |  |  |  |  |

*ANCOVA:*

Significant effects were found for both, primary and secondary outcome measures following completer analysis: CBI *F*(1, 51)=7.68, *P*= .008; PHQ-9 *F*(1, 51)=8.24, *P*= .006; GAD-7 *F*(1, 51)=12.84, *P*= .001; PSS-14 *F*(1, 51)=24.01, *P*< .001; BBQ *F*(1, 51)=16.43, *P*< .001; WHO-5 *F*(1, 51)=17.44, *P*< .001. Significant changes for subscales of Development and Physical Health were also observed in the completer sample, *F*(1, 51)=11.05, *P*= .002 and *F*(1, 51)=8.62, *P*= .005 respectively, with addition of significant findings in the subscale of the Social Relationships, *F*(1, 51)=4.50, *P*= .04. No significant changes were detected for Emotional Health and Time Dependency subscales, *F*(1, 51)=2.68, *P*= .11 and *F*(1, 51)= .30, *P*= .59 respectively.

*Clinically significant change and RCI of caregiver burden*

In the completer sample McNemar's test has also displayed a significant difference between the groups, *P*=0.01. Regarding clinical significance, in the intervention group 2 (8%) participants scored below cut-off score of 28.8. In the control group, 1 (3.4%) participant was found to achieve clinically significant change. RCI results are presented in the table 6.

Table 6 *RCI of participants for completer sample*

|  | RCI for CBI completer sample | | |
| --- | --- | --- | --- |
|  | Positive RCI | No change | Negative RCI |
| Intervention group: n (%) | 12 (48.0%) | 10 (40.0%) | 3 (12.0%) |
| Wait list control group: n (%) | 2 (6.9%) | 22 (75.9%) | 5 (17.2%) |
